# A modified surgical technique of shortening renal ischemia time in left renal cancer patients with Mayo level II-IV tumor thrombus

**DOI:** 10.1186/s12893-020-00769-w

**Published:** 2020-06-05

**Authors:** Liu Zhuo, Zhu Guodong, Zhao Xun, Tang Shiying, Hong Peng, Zhang Li, Li Liwei, Zhang Shudong, Wang Guoliang, Tian Xiaojun, Liu Cheng, Zhang Hongxian, Ma Lulin

**Affiliations:** 1 Urology Department of Peking University Third Hospital, Beijing, 100083 China; 2 Ultrasound Diagnosis Department of Peking University Third Hospital, Beijing, 100083 China

**Keywords:** Inferior vena cava, Left side, Renal cancer, Renal ischemia, Tumor thrombus

## Abstract

**Background:**

To explore the safety and effectiveness of a modified surgical technique which could shorten the time of renal ischemia in left renal cancer and Mayo level II to IV inferior vena cava (IVC) tumor thrombus.

**Method:**

We retrospectively analyzed the clinical data of 14 cases with left renal cell carcinoma (RCC) and Mayo level II to IV IVC tumor thrombus from February 2015 to July 2019. Preoperative imaging showed that there was no obvious sign of tumor thrombus invading the blood vessel wall. During the surgery, after the right renal artery, the right renal vein and the distal end of IVC were blocked, the balloon catheter was used and the tumor thrombus was removed completely from the IVC. The incision of IVC was closed by Satinsky clamp to make IVC partially blocked. Then the right renal artery and right renal vein were released. The incision of IVC was sutured continuously. At last, the Satinsky clamp and the blocking band at the distal end of the IVC were released.

**Result:**

There were 8 cases (57.1%) of Mayo level II, 3 cases (21.4%) of Mayo level III and 3 cases (21.4%) of Mayo level IV. The operation was successfully completed in all 14 patients. There were 2 cases (14.3%) operated by complete laparoscopic approach, 8 cases (57.1%) by open approach, and 4 patients (28.6%) by laparoscopic conversion to open approach. The occlusion time of right renal artery and vein (renal ischemia time) was 3 to 15 min, with an average of (6.8 ± 3.2) minutes. The mean time of IVC occlusion was (19.4 ± 4.9) min. Preoperative creatinine was 66 to 130 μmol/L, with an average of (96.6 ± 21.2) μmol/L. One week after operation, serum creatinine was 64 to 632 μmol/L, with an average of (132.4 ± 144.9) μmol/L. Among the 14 cases, 5 (42.9%) had early postoperative complications. Besides one of the 14 patients died in perioperative period, the median follow-up of other 13 cases was 10 months (range: 4–29 months). The 5 (35.7%) of the 14 cases were died of disease.

**Conclusion:**

This modified procedure was relatively safe and effective in shortening the time of renal ischemia in left RCC patients with Mayo II to IV IVC tumor thrombus.

## Background

Inferior vena caval tumor thrombus (IVC-TT) occurs in 10% of patients diagnosed with renal cell carcinoma (RCC) [1]. Because of different anatomical characteristics, compared with the right RCC, the left RCC patients with inferior vena cava (IVC) tumor thrombus are more likely to have renal dysfunction after operation [1]. For right RCC with IVC tumor thrombus, the healthy left renal artery was not necessary to be blocked during the operation. Only the left renal vein is temporarily blocked in the procedure of thrombectomy. The venous blood flow of the left kidney can pass through the collateral circulation mainly composed of the ascending lumbar vein. For patients with left RCC and IVC tumor thrombus, the healthy right kidney usually does not have sufficient collateral circulation [2]. The blood of the left kidney mainly flowed back through the collateral circulation to prevent renal congestion. These collateral circulations included ascending lumbar vein, adrenal vein, gonadal vein, ureteral vein, inferior phrenic vein, the second and third lumbar vein and renal capsule vein. But for left RCC, the right kidney usually did not have enough collateral circulation. The right kidney could only rely on the unsteady gonadal vein, small ureteral vein and renal capsule vein for reflux. During the operation, the right renal artery should also be blocked to prevent renal congestion [3]. However, the long-time blocking of right renal artery and vein would affect the renal function after operation [4]. Therefore, how to improve the surgical technique to shorten the time of right renal ischemia is of great significance.

The traditional operation method is to fully expose the right renal artery, right renal vein, the distal and proximal end of IVC and then block them in a certain order. First, the distal end of IVC was blocked, Second, the right renal artery, Third, the right renal vein, Fourth, the proximal end of IVC. After the IVC wall was cut and the tumor thrombus was resected, the above vessels were released in turn [5]. First, the proximal end of IVC were released, Second, the right renal vein, Third, the right renal artery, Fourth, the distal end of IVC. The limitation of this procedure is that the right renal artery and right renal vein are blocked for a long time, which is almost equal to the total time of IVC block, including the time of complex vascular suture.

Previous studies have reported the advantage of using Satinsky clamp to partially block the IVC in the surgical procedure of left RCC with Mayo I IVC tumor thrombus. Our study goes further to make this technique improved and applied to Mayo II to IV IVC tumor thrombus patients to shorten the time of renal ischemia during the operation. We retrospectively analyzed the clinical data of 14 cases with left RCC and Mayo level II to IV IVC tumor thrombus, aiming to explore the safety and effectiveness of this modified surgical technique.

## Methods

### General information

Fourteen cases were included in this study. We collected the data of the patients’ clinical manifestations, including local symptoms such as hematuria, lumbago, abdominal mass, etc.) or systemic symptoms (such as emaciation, fever, fatigue, anemia, etc.). All patients should underwent several series of imaging examination. The clinical stage and Mayo level of IVC tumor thrombus were determined by enhanced CT scan of urinary system [6]. Color Doppler ultrasound could effectively detect the frequency spectrum of the vessels in the tumor thrombus, and help to judge the degree of IVC obstruction by blood perfusion information. Echocardiography could be used to judge the right atrial tumor thrombus for the choice of surgical methods and the evaluation of operation risk. Intraoperative transesophageal ultrasound was used to determine the location of the proximal end of the tumor thrombus in patients with Mayo III or IV tumor thrombus. MRI enhancement could indicate the length of tumor thrombus and whether the IVC wall was invaded by tumor thrombus [7]. Other imaging examination items included: chest CT, radionuclide renogram, radionuclide bone imaging for patients with bone pain, MRI scanning for patients with central nervous system symptoms and PET-CT. The recommended laboratory tests included preoperative and postoperative creatinine, hemoglobin, albumin and total protein. We used the American Society of anesthesiologists (ASA) score to assess the anesthesia risk [8] and Mayo classification to evaluate the level of tumor thrombus in renal vein or IVC [9]. Clinical staging of tumors was classified by TNM staging (2010 International Union of anticancer, UICC).

The inclusion criteria of patients in this study was as follows:① CT and/or MRI showed that the left renal malignant tumor was accompanied by IVC tumor thrombus; ② The tumor thrombus of IVC was classified as Mayo level II to IV; ③ Preoperative imaging showed that there was no obvious sign of tumor thrombus invading the blood vessel wall. In the enhanced MRI examination, the direct sign of tumor thrombus invading the blood vessel wall includes the tumor thrombus strengthening and breaking through the blood vessel wall, resulting in tumor signals displayed on both sides of the blood vessel wall; ④ The histology was RCC.

IVC enhanced MRI was performed in all 14 patients with tumor thrombus. The direct signs of invasion of inferior vein wall by tumor thrombus in preoperative MRI were as follows: In contrast-enhanced examination, both the medial and lateral walls of inferior vena cava were enhanced. Indirect signs include: ① The wall of inferior vena cava was rough and unsmooth with “burr sign”; ②The diameter of inferior vena cava increased; ③ Edema zone could be seen on the lateral wall of inferior vena cava; ④The shape of tumor thrombus in inferior vena cava was irregular. In this study, there were no direct or indirect signs of tumor thrombus invading the blood vessel wall in 14 patients with tumor thrombus. The height of tumor thrombus was in the form of Mayo grade, as shown in the table.

### Surgical procedure

**Fig. 1:**
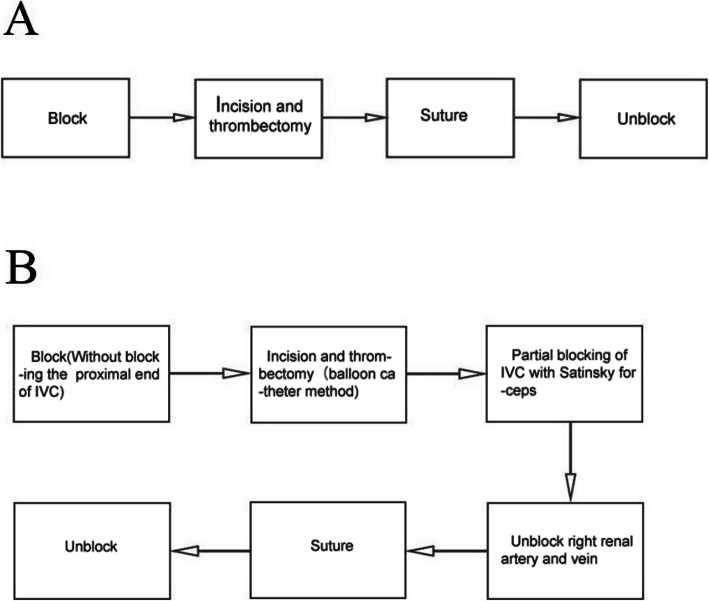
The difference between the traditional operation method and the modified operation method procedures in left renal cancer and Mayo level II to IV inferior vena cava thrombectomy. **a** the traditional operation method; **b** the modified operation method

The radical nephrectomy was performed by laparoscopic or open approach [10]. The steps were as follows: the left renal artery and ureter were separated and ligated. The left kidney was dissociated outside the perirenal fascia, only the left renal vein was connected to the IVC [11]. The traditional operation method and the modified operation method procedures were shown in Fig. 1.

As for the choice of laparoscopic or open surgical approach, we would consider a variety of factors. For example: ① The diameter of primary renal tumor: For patients with smaller tumor diameter, there was enough space to use the retroperitoneal approach. ② The height of tumor thrombus: For most of Mayo II tumor thrombus, we mostly adopt laparoscopic approach, while for Mayo III or Mayo IV tumor thrombus, the liver needed to be free, and the intrahepatic inferior vena cava needed to be exposed. Open approach was often chosen. ③ Patient factors: Open approach was often chosen to treat the elderly patients or patients with previous pulmonary basic diseases to avoid acidosis caused by carbon dioxide pneumoperitoneum. In addition, we also needed to take into account such factors as the experience of the surgeon, the degree of adhesion between the tumor and the surrounding tissue, etc. The advantages of minimally invasive surgery such as laparoscopy could not be at the expense of efficacy and patient safety. In case of severe adhesions, difficulties in operation, increased bleeding and other emergencies, the laparoscopic approach should be decisively converted to open surgery.

Robot surgery was expensive and most of the patients with renal cancer associated with IVC tumor thrombus came from areas with poor economic conditions. Therefore, patients choosed more open surgery or laparoscopic surgery with low price. Another important reason was that our center had not yet carried out robot surgery, and we lacked robot related surgical instruments. That’s why we don’t use a robotic approach in the research.

For patients with left renal tumor and tumor thrombus, the left retroperitoneal approach combined with transperitoneal approach could be used. The patient was placed in a right lateral position with the lumbar bridge elevated. To establish the retroperitoneal space, a 2 cm longitudinal incision was performed at the twelfth costal margin at the leading edge of psoas. The fingers were used to separate and expand the retroperitoneal space. The balloon was implanted, 400-600 ml of air was injected and the dilation lasted for 5 min. A 13 mm trocar was inserted. Carbon dioxide pneumoperitoneum was established and maintained at 12 mmHg. An 11 mm trocar was inserted above the iliac crest in the axillary midline. A 5 mm trocar was inserted under the costal margin in the axillary front line. If necessary, A 5 mm trocar could be inserted about 5 cm inside the anterior superior iliac spine to assist. In the left retroperitoneal approach, the left renal artery was dissociated and cut off; the left ureter was ligated; the adrenal gland was explored; the left kidney was completely dissociated except the left renal vein. The left peritoneum was opened along the paracolon sulcus to facilitate subsequent removal of the kidney. A perirenal drainage tube was left and the incisions were sutured.

Transperitoneal approach was used for conversion: The patient was placed in a left lateral position with the lumbar bridge elevated. Four trocars were placed. The location was as follows: 1. An 11 mm trocar was placed beside the right rectus abdominis muscle, at the level of umbilicus. Pneumoperitoneum was connected and 30 ° laparoscopy was introduced; 2. A 13 mm trocar was placed 3-5 cm above the anterior superior iliac spine; 3. A 5 mm trocar was placed 3–5 cm below the costal margin of the axillary midline under direct vision to avoid the injury of intercostal artery. The ultrasonic knife and forceps were respectively placed. 4. A 5 mm trocar was placed 1 cm below the xiphoid process and a trefoil clamp was placed to expose the liver. The colon and duodenum were freed, the IVC was completely freed, and the right renal artery and right renal vein were freed to be blocked. The IVC was dissociated to the hepatic level, and the initial segment of the left renal vein was dissociated. The wall of IVC was cut and the tumor thrombus was removed. The incision of IVC was sutured and the blockage was relieved.

Chevron incision was selected for open surgery. For left RCC, the incision was chosen 2 cm below the left costal margin, from xiphoid process to axillary midline, and extended about 10 cm to the right costal margin. Other surgical procedures were described above.

**Fig. 2:**
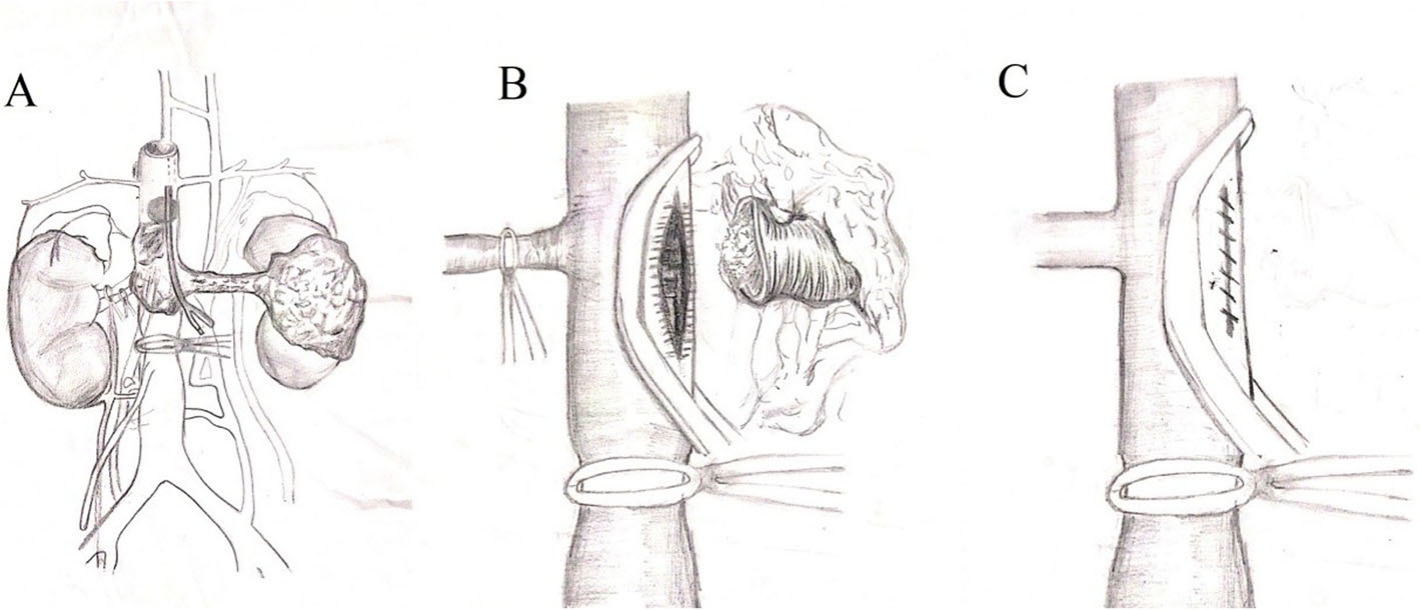
The procedure of the modified operation method: **a** The right renal artery, the right renal vein and the distal end of IVC were twined by double circles of vascular blocking tape; A small incision of 0.5 cm was made with the scalpel. The balloon catheter was placed into the small incision and extended to the proximal end of tumor thrombus. **b** Pull the balloon catheter and remove the tumor thrombus completely from the IVC. The incision of IVC was clipped with Satinsky clamp, and the IVC was partially blocked. **c** The right renal artery and right renal vein were unblocked. The incision of IVC was sutured continuously with non-absorbable vascular suture

The procedure of the modified operation method was as follows (shown in Fig. 2): ① The right renal artery, right renal vein, and the distal and proximal ends of IVC were fully exposed. The right renal artery, the right renal vein and the distal end of IVC were twined by double circles of vascular blocking tape, as the blocking time was recorded. ② At the entrance of the left renal vein into the IVC, a small incision of 0.5 cm was made with the scalpel. The balloon catheter was placed into the small incision and extended to the proximal end of tumor thrombus (Fig. 2a). Finger palpation or transesophageal ultrasonography was used to ensure that the end of the balloon catheter crossed the proximal end of the tumor thrombus. The assistant used a syringe to inject water into the balloon to expand it. Finger palpation was performed again to determine the balloon completely blocked the proximal end of IVC.③ The small incision of 0.5 cm was extended up to 2.5-5 cm parallel to the long axis of IVC with vascular scissors. Pull the balloon catheter and remove the tumor thrombus completely from the IVC. We used transesophageal ultrasonography to confirm that the tumor thrombus was completely removed. ④ The incision of IVC was clipped by Satinsky clamp, and the IVC was partially blocked (Fig. 2b). Then the right renal artery and right renal vein were unblocked. The blocking time of renal ischemia was recorded. ⑤ The incision of IVC was sutured continuously with non-absorbable vascular suture (Fig. 2c). The IVC was flushed with heparin saline to make the IVC full and discharge air to avoid air embolism. At the end, the Satinsky clamp used to partially block the IVC was removed, and the blocking band at the distal end of the IVC was removed. The blocking time of IVC was recorded.

### Postoperative complications and follow-up

In terms of complications, Clavien grading system was used to count the incidence, type, treatment and prognosis of complications [12]. In the types of complications, we focused on the occurrence of renal insufficiency. In the evaluation of the difficulty of operation and prognosis, the blocking time of right renal artery and right renal vein (the time of renal ischemia), the blocking time of IVC, the total operating time, the amount of intraoperative hemorrhage, the volume of intraoperative transfusion of suspended red blood cells, and the postoperative hospital stay were counted. The patients were followed up every 3 months for the first 2 years, every 6 months until year 5, and annually thereafter. The main contents of follow-up examination include blood biochemistry test, abdominal B-ultrasound and/or urinary system enhanced CT, chest film or chest enhanced CT, etc.

## Results

This study included 14 patients. Nine cases were male, and five cases were female. The average age is (62.1 ± 8.7) years (48–76 years). The mean body mass index (BMI) was (22.6 ± 2.2) kg/m^2^ (19.1 to 26.0 kg/m^2^). In terms of clinical manifestations, there were 1 case (7.1%) without symptoms, 4 cases (28.6%) with local symptoms, 3 cases (21.4%) with systemic symptoms, and 6 cases (42.9%) with both local symptoms and systemic symptoms. There were 11 cases (78.6%) with ASA score of 2 and 3 cases (21.4%) with ASA score of 3. The tumor diameter was 5.0 to 21.1 cm, with an average of (9.2 ± 3.9) cm. In clinical stage, there were 4 cases (28.6%) of N0 and 10 cases (71.4%) of N1. There were 10 cases (71.4%) with M0 and 4 cases (28.6%) with M1. There were 8 cases (57.1%) of Mayo level II, 3 cases (21.4%) of Mayo level III and 3 cases (21.4%) of Mayo level IV. The preoperative average hemoglobin was 107.9 ± 23.5 g/L (73 to 150 g/L), the average albumin was 37.7 ± 5.6 g/L (28 to 51 g/L), and the average total protein was 69.0 ± 8.9 g / L (43 to 80 g/L), as shown in Table 1.

**Table 1:** Clinical features of patients with application of a modified operation shorten the time of renal ischemia

Patients (n)	14
mean value ± SD (min-max)	
Age, years	62.1 ± 8.7 (48–76)
BMI, kg/m^2^	22.6 ± 2.2 (19.1–26.0)
Tumor diameter, cm	9.2 ± 3.9 (5.0–21.1)
Albumin, g/L	37.7 ± 5.6 (28–51)
Total Protein, g/L	69.0 ± 8.9 (43–80)
Hemoglobin, g/L	107.9 ± 23.5 (73–150)
N (%)	
Sex	
Male	9 (64.3%)
Female	5 (35.7%)
ASA score	
2	11 (78.6%)
3	3 (21.4%)
Clinical symptoms	
No clinical symptoms	1 (7.1%)
Local symptoms	4 (28.6%)
Systemic symptoms	3 (21.4%)
Both	6 (42.9%)
cN stage	
cN0	4 (28.6%)
cN1	10 (71.4%)
cM stage	
cM0	10 (71.4%)
cM1	4 (28.6%)
Mayo classification	
II	8 (57.1%)
III	3((21.4%)
IV	3((21.4%)

The operation was successfully completed in all 14 patients, and there was no death during the operation. There were 2 cases (14.3%) performed by complete laparoscopic approach, 8 cases (57.1%) by open approach, and 4 patients (28.6%) by laparoscopic conversion to open approach. The occlusion time of right renal artery and vein (renal ischemia time) was 3 to 15 min, with an average of (6.8 ± 3.2) min. The occlusion time of IVC was 12 to 30 min, with an average of (19.4 ± 4.9) min. The total operation time was 340 to 589 min, with an average of (454.0 ± 92.0) min. The average intraoperative bleeding volume was (2067.9 ± 1373.6) ml.

Four cases were performed by laparoscope conversion to open approach actively or passively. In 1 case, it was difficult to separate and expose under laparoscope because of the adhesion of renal tumor and surrounding tissue. In 1 case, the adhesion between renal vein or IVC and the surrounding tissue outside the vessel wall was serious. In 2 cases, radical nephrectomy was performed by laparoscopy, and tumor thrombectomy was removed by open approach in active conversion.

**Fig. 3:**
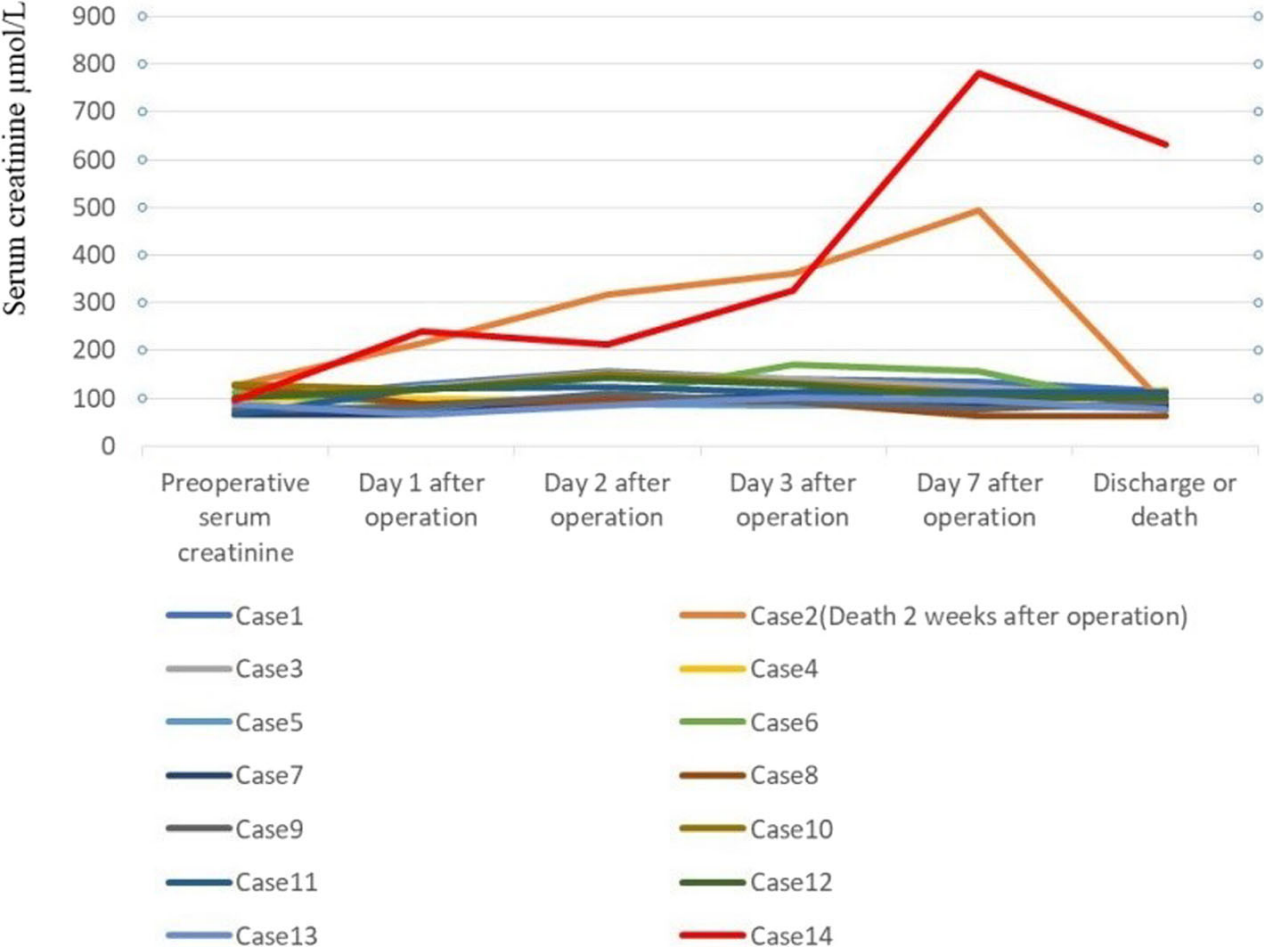
The change value of serum creatinine during perioperative period in patients with left renal cancer and Mayo level II to IV inferior vena cava tumor thrombus

The average transfusion volume of red blood cells was (1592.9 ± 1118.0) ml. The average transfusion volume of plasma was (538.5 ± 670.3) ml. The average postoperative hospital stay was (12.9 ± 6.2) days. Preoperative creatinine was 66 to 130 μmol/L, with an average of (96.6 ± 21.2) μmol/L. One week after operation, serum creatinine was 64 to 632 μmol/L, with an average of (132.4 ± 144.9) μmol/L as shown in Table 2. The change value of serum creatinine in perioperative period was shown in Fig. 3. Only 2 patients (14.3%) had postoperative renal insufficiency. The renal function of 12 patients (85.7%) recovered to the preoperative level. There were 12 cases (85.7%) with clear cell carcinoma, 1 case (7.1%) with papillary carcinoma and 1 case (7.1%) with undifferentiated type. There were 5 cases (35.7%) in WHO/ISUP 2016 pathological grade 2, 6 cases (42.9%) in grade 3, 3 cases (21.4%) in grade 4.

**Table 2:** Perioperative data and postoperative pathology of patients with application of a modified operation shorten the time of renal ischemia

Patients (n)	14
mean value ± SD (min-max)	
Preoperative serum creatinine,μmol/L	96.6 ± 21.2 (66–130)
serum creatinine one week after operation,μmol/L	132.4 ± 144.9 (64–632)
Warm ischemia time (WIT) for right kidney (artery and vein),min	6.8 ± 3.2 (3–15)
IVC clamping time, min	19.4 ± 4.9 (12–30)
Operative time,min	454.0 ± 92.0 (340–589)
Surgical bleeding volume,ml	2067.9 ± 1373.6 (50–5000)
Surgical blood transfusion volume,ml	1592.9 ± 1118.0 (0–3200)
Plasma transfusion volume,ml	538.5 ± 670.3 (0–2000)
Postoperative hospital stay	12.9 ± 6.2 (6–25)
N (%)	
Operative method	
Complete laparoscopic approach	2 (14.3%)
Laparoscopic transfer to open approach	4 (28.6%)
Open approach	8 (57.1%)
Pathology type	
Clear cell carcinoma	12 (85.7%)
Papillary carcinoma	1 (7.1%)
Undifferentiated type	1 (7.1%)
WHO/ISUP2016 pathological grading	
2	5 (35.7%)
3	6 (42.9%)
4	3 ((21.4%)
Postoperative complications	
No	9 (64.3%)
Yes	6 (35.7%)

**Fig. 4:**
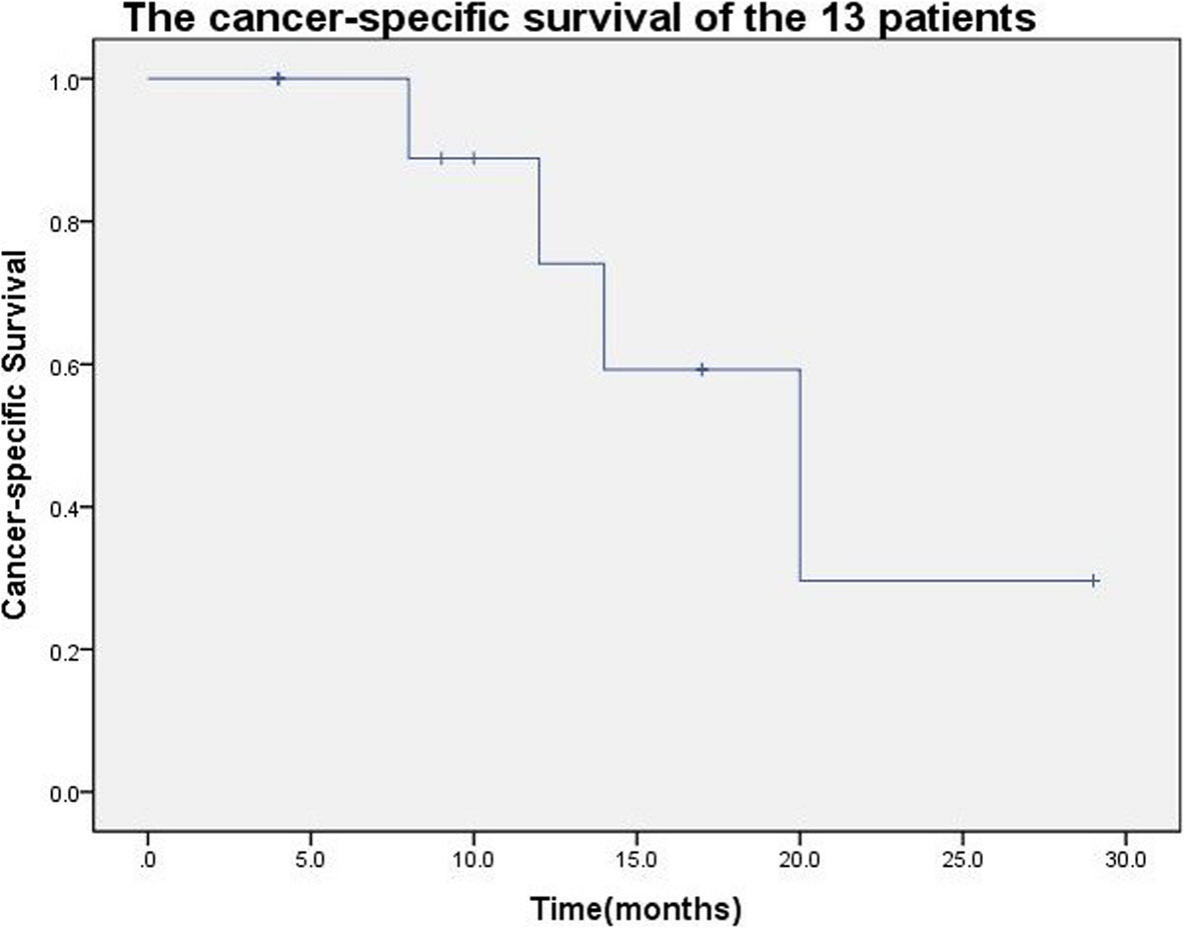
Survival curve of 13 patients with left renal cancer and Mayo level II to IV inferior vena cava tumor thrombus

Among the 14 cases, 5 (42.9%) had early postoperative complications. There was 1 case with Clavien system grade I, whose clinical manifestation was atelectasis, and the patient improved after conservative treatment. Clavien system grade II complications occurred in 2 cases. One case showed chylous fistula in drainage fluid, and the patient improved after restriction of feeding, intravenous rehydration and somatostatin treatment; Another patient developed central venous catheter-related infection and bilateral pleural effusion, and the patient improved by antibiotic treatment. There was 1 case with Clavien system grade IVa, whose clinical manifestation was renal insufficiency and he was treated with hemofiltration. Clavien system grade V complications occurred in 1 case. The patient underwent cardiopulmonary bypass during operation, and developed hypoxemia, septic shock, multiple organ dysfunction including renal insufficiency, coagulation dysfunction and died 14 days after operation. One of the 14 patients died in perioperative period. The other 13 cases were followed up for 4 to 29 months, and the median follow-up was 10 months. The mean survival time was 19.3 ± 3.0 months, and the median survival time was 20 months. Tumor specific death occurred in 5 (35.7%) of the 14 cases, as shown in Fig. 4.

## Discussion

The improvement of this technique was based on the advantage of Satinsky clamp to partially block the IVC in the operation of left RCC with Mayo level I tumor thrombus. The technique was improved and applied to patients with Mayo II to IV tumor thrombus to shorten the time of renal ischemia. The key to the surgical treatment of Mayo I tumor thrombus was that the proximal end of the tumor thrombus was located near the place where the renal vein entered the IVC. Satinsky clamp was used to partially block the IVC, so as to ensure that the blood flow of the right renal vein, the distal and proximal end of the IVC was partially unobstructed. Its advantage was that there was no need to block the right renal artery and vein to prevent renal ischemia and protect renal function. However, the limitation of its application was that the distal end of the tumor thrombus should be located near the entrance of renal vein, which was not suitable for Mayo II tumor thrombus which meant the tumor thrombus invaded more than 2 cm from the entrance into IVC. For left RCC with Mayo II tumor thrombus, the traditional method was to fully expose the right renal artery, renal vein, distal and proximal end of IVC. The above vessels were blocked by vascular blocking band. The incision of IVC was long. Its advantage was that the IVC segment had no blood flow after the related blood vessels were fully blocked, so as to control the bleeding to the greatest extent. But its limitation was that the time of right renal artery and vein occlusion was longer, almost equal to the total time of IVC occlusion, which included the time of blood vessel suture. In this group of patients, the average occlusion time of right renal artery and vein (renal ischemia time) was 6.8 min. The average time of IVC occlusion was 19.4 min. We believed that the time of right renal ischemia could be reduced by separating the steps of renal portal occlusion and IVC vascular suture.

The improved technique was suitable for the patients whose tumor thrombus did not invade the blood vessel wall. For this kind of patients, the balloon catheter could be used to reduce the difficulty of operation. In the previous study, we described in detail the procedure of balloon catheter [13]. The IVC incision did not need to be too long, and the tumor thrombus could be removed by allowing the dilated balloon to pass through. Therefore, the small incision provided the possibility for Satinsky clamp to partially block the IVC.

Because of the different anatomic characteristics between the left and the right kidney, the left RCC patients with IVC tumor thrombus were more likely to have renal dysfunction. For the right RCC, it was necessary to cut the wall of IVC after blocking the distal end of IVC, the left renal vein and the proximal end of IVC, while the left renal artery was usually not blocked. The blood of the left kidney mainly flowed back through the collateral circulation to prevent renal congestion. Therefore, in addition to the distal end of IVC, the left renal vein and the proximal end of IVC, the right renal artery must be blocked to prevent renal congestion. The long-time blocking of right renal artery and vein would affect the renal function after operation. Therefore, how to shorten the time of right renal ischemia was the key of the operation. In the procedure of incision and suture of the blood vessel wall, the IVC was partially blocked by Satinsky clamp, and the right renal portal vessel was removed first. It shortened the time of right renal ischemia. In our study, only 2 patients (14.3%) suffered postoperative renal insufficiency. One case with Mayo IV tumor thrombus had septic shock and multiple organ dysfunction including renal insufficiency. The other patient was with Mayo III tumor thrombus treated with hemodialysis. The renal function of the rest 12 patients (85.7%) recovered to the preoperative level.

This technology also has some limitations. It was not suitable for patients with tumor thrombus invading the blood vessel wall. For these patients, there might be adhesion between tumor thrombus and the IVC [14] and it was not suitable to take out the thrombus by balloon catheter. Otherwise, it might lead to failure of thrombus removal, residual tumor thrombus, falling off of tumor thrombus or even pulmonary embolism and other serious complications. Therefore, preoperative imaging judgment was important. MRI scan could be used to judge whether the tumor thrombus invaded the IVC wall. The sensitivity, specificity and accuracy of MRI were 100, 89 and 92%, respectively [15]. The direct sign of vein wall invasion was enhancement of tumor thrombus and breakthrough of vessel wall, which resulted in tumor signal emerged on both sides of vessel wall [7]. Indirect signs include: ①Rough and unsmooth wall of IVC with “burr sign” [7];②The diameter of IVC increased obviously [16, 17];③There was edema zone on the lateral side of IVC;④The shape of tumor thrombus was irregular. Although preoperative imaging could be used to judge whether the tumor thrombus invaded the IVC wall to some extent, intraoperative visual diagnosis was still an accurate and effective method. The signs were as follows: the wall of blood vessel was rough, unsmooth and white; the texture of palpation was hard and the elasticity of blood vessel was poor. Appropriate patient selection was the key to the success of this technique.

This study has the following limitations: (1) This was a retrospective study and its conclusions need to be confirmed in a prospective controlled study; (2) The sample size of this study was small. The reason was that the right renal vein (about 2-3 cm long) was shorter than the left (6-7 cm long), and the tumor thrombus was more likely to surpass the renal vein and invade into the IVC in right RCC than the left. Therefore, the incidence rate of left renal cell carcinoma with Mayo II was lower than that of the right side. (3) The median follow up of the 13 patients was only 10 months, with an interval between 4 to 29 months: a median follow up < 12 months limits the oncologic survival outcomes. Therefore, large sample size and long-term follow-up data are still needed.

The following limitations still existed in this study. The number of cases in this group was too small and more cases were needed to verify the results. Besides the results of this study were maybe afflicted by the presence of 8 (57%) cases of Mayo level II thrombus which were clearly easier to treat than type III and IV. The height of tumor thrombus had influence on the difficulty of operation.

## Conclusion

This modified procedure was relatively safe and effective in shortening the time of renal ischemia in left RCC patients with Mayo II to IV IVC tumor thrombus.

## Data Availability

The analysed data sets generated during the study are available from the corresponding author upon reasonable request.

## Abbreviations

IVC: Inferior vena cava
RCC: Renal cell carcinoma
CT: Computed tomography
MRI: Magnetic resonance imaging
PET-CT: Positron emission tomography-computed tomography
ASA: American Society of anesthesiologists
